# Diagnosis of late-onset Pompe disease and other muscle disorders by next-generation sequencing

**DOI:** 10.1186/s13023-016-0390-6

**Published:** 2016-01-25

**Authors:** Sébastien Lévesque, Christiane Auray-Blais, Elaine Gravel, Michel Boutin, Laura Dempsey-Nunez, Pierre-Etienne Jacques, Sébastien Chenier, Sandrine Larue, Marie-France Rioux, Walla Al-Hertani, Amelie Nadeau, Jean Mathieu, Bruno Maranda, Valérie Désilets, Paula J. Waters, Joan Keutzer, Stephanie Austin, Priya Kishnani

**Affiliations:** Department of Pediatrics, Division of Medical Genetics, Faculty of Medicine and Health Sciences, Université de Sherbrooke, and Centre Hospitalier Universitaire de Sherbrooke, 3001, 12th Avenue North, Sherbrooke, QC J1H 5N4 Canada; Departments of Biology and Computer Science, Faculty of Sciences, Université de Sherbrooke, Sherbrooke, QC Canada; Department of Neurology, Notre-Dame Hospital, Université de Montréal, Montreal, QC Canada; Department of Neurology, Université de Sherbrooke, and Centre Hospitalier Universitaire de Sherbrooke, Sherbrooke, QC Canada; Department of Pediatrics, Cumming School of Medicine, University of Calgary, and Alberta Children’s Hospital, Calgary, AB Canada; Department of Pediatrics, Division of Pediatric Neurology, Université de Sherbrooke, and Centre Hospitalier Universitaire de Sherbrooke, Sherbrooke, QC Canada; Neuromuscular Clinic, Centre de réadaptation en déficience physique de Jonquière, Saguenay, QC Canada; Genzyme Corporation, a Sanofi Company, Cambridge, MA USA; Department of Pediatrics, Division of Medical Genetics, Duke University Medical Center, Durham, NC USA

**Keywords:** Pompe disease, Lysosomal disorders, Muscle disorders, Next-generation sequencing

## Abstract

**Background:**

Late-onset Pompe disease (LOPD) is a rare treatable lysosomal storage disorder characterized by progressive lysosomal glycogen accumulation and muscle weakness, with often a limb-girdle pattern. Despite published guidelines, testing for LOPD is often overlooked or delayed in adults, owing to its low frequency compared to other muscle disorders with similar muscle patterns. Next-generation sequencing has the capability to test concurrently for several muscle disorders. This could potentially lead to increased diagnosis of LOPD, disorders with non-specific muscle weakness or atypical patients.

**Methods:**

We developed a gene panel to further study its clinical utility in a cohort of patients with suspected muscle disorders. We designed a gene panel to analyze the coding sequences and splice site junctions of *GAA* causing LOPD, along with 77 other genes causing muscle disorders with overlapping phenotypes.

**Results:**

At a median coverage of ~200X (sequences per base), all *GAA* exons were successfully covered with >20X and only 0.3 % of exons across all genes were <20X. The panel showed an excellent sensitivity (100 %) and specificity (98 %) across all selected genes, using known variations in Pompe patients and controls. We determined its clinical utility by analyzing 34 patients with suspected muscle disorders of undetermined etiology and various muscle patterns, who were referred or followed in neuromuscular and genetics clinics. A putative diagnosis was found in up to 32 % of patients. The gene panel was instrumental in reaching a diagnosis in atypical patients, including one LOPD case. Acid alpha-glucosidase activity was used to confirm the molecular results in all patients.

**Conclusion:**

This work highlights the high clinical utility of gene panels in patients with suspected muscle disorders and its potential to facilitate the diagnosis of patients showing non-specific muscle weakness or atypical phenotypes. We propose that gene panels should be used as a first-tier test in patients with suspected muscle disorders of undetermined etiology, which could further increase overall diagnosis of muscle conditions, and potentially reduce diagnostic delay. Further studies are necessary to determine the impact of first-tier gene panels on diagnostic delay and on treatment outcome for LOPD.

**Electronic supplementary material:**

The online version of this article (doi:10.1186/s13023-016-0390-6) contains supplementary material, which is available to authorized users.

## Background

Pompe disease (OMIM 232300) is an autosomal recessive condition caused by mutation in the *GAA* gene, leading to a deficiency in acid alpha-glucosidase (OMIM 606800, EC 3.2.1.20) and accumulation of glycogen in all tissues, most notably in muscles and heart [1, 2]. Pompe disease consists of a phenotypic continuum, however two broad categories are recognised: classic infantile and late onset forms [3, 4]. Classical infantile Pompe disease presents in the first few months of life with hypertrophic cardiomyopathy, generalized muscle weakness, and respiratory distress [5]. Without enzyme replacement therapy (ERT), the disease is fatal within the first year of life in the vast majority of infantile cases [6]. Late-onset Pompe disease (LOPD) may present at any age from late infancy onwards, but most frequently develops during adulthood, either with motor delays or limb-girdle muscle weakness [7]. Some patients present with rigid spine syndrome, scoliosis and low body mass [7]. Diaphragmatic weakness is present in the vast majority of patients and leads to nocturnal hypoventilation. Presymptomatic patients can be identified by persistent, chronic elevation of serum creatine kinase, and a recent study showed a frequency of Pompe disease in 2.5% of patients with chronic hyperCKemia [8]. The combined incidence of all forms of Pompe disease at birth is estimated to be 1:40,000 in the US and similarly in Netherlands, with the adult onset form being 1:57,000 [9, 10]. However, recent data from newborn screening program of Missouri state, Austria and Taiwan suggest that incidence is higher with respectively 1:5463, 1:8684 and 1:11,987 [11–13]. LOPD is likely underdiagnosed in neuromuscular clinics owing to the rarity of the condition and its marked phenotypic variability.

Since early diagnosis, followed with prompt initiation of ERT, may significantly improve its clinical benefits, universal screening has been advocated as a way to improve outcomes in Pompe patients [14]. For LOPD, delayed diagnosis is not unusual and has been reported to be made almost 10 years after the onset of symptoms [15]. Pilot newborn screening programs are in development or in progress in the US, and are well established in Taiwan using dried blood spots [16–18]. Although LOPD cases can be detected by newborn screening, undiagnosed children and adults will not benefit from ongoing development of these screening programs. Current methodologies to confirm the diagnosis of Pompe include mutation analysis by Sanger sequencing and enzyme testing in fibroblasts or leukocytes using fluorimetric methods, or more recently, on dried blood spot by tandem mass spectrometry [4, 19, 20]. All these methods are contingent on a strong clinical suspicion of Pompe disease and do not detect the presence of other muscle disorders. Given the rarity of Pompe disease and despite published diagnostic guidelines to educate clinicians, testing for Pompe disease is often overlooked [4]. The need exists for more comprehensive diagnostic tools targeting Pompe disease among other muscle disorders with overlapping clinical manifestations. The differential diagnosis of Pompe disease includes many genetic muscle disorders with limb-girdle weakness or rigid spine syndrome, which may be difficult to differentiate on a clinical basis alone [4]. However, next-generation sequencing (NGS) has the capability to test concurrently for all these muscle disorders.

NGS technologies make it possible to perform several millions of accurate sequence reactions in parallel on solid support, each producing a short sequence called a “read” [21]. Sequence enrichment methods are available to limit the analysis to the exons of selected genes of interest (gene panels). Pitfalls of sequence enrichment include low number of sequence reads in GC-rich regions or very low GC content, which would need to be sequenced by the conventional Sanger method to prevent false negatives [22, 23].

We developed a gene panel to simultaneously analyze genes associated with several muscle disorders with overlapping phenotypes in order to increase the diagnosis of LOPD in children and adults, of other disorders with non-specific muscle patterns and of patients with atypical presentations. We validated this gene panel using CEPH cell line NA12878 and samples from known Pompe patients and determined its clinical validity among a group of patients with undetermined muscle disorders. In parallel, we validated a method to determine acid alpha-glucosidase activity on dried blood spots (DBS) by tandem mass spectrometry, and correlated the enzyme activity data with results from the gene panel.

## Methods

### Gene panel design

We included genetic disorders that are part of the differential diagnosis of LOPD [4, 24]: muscular dystrophies with limb-girdle weakness pattern, rigid spine syndromes, scapuloperoneal syndromes, congenital myasthenic syndromes, congenital myopathies (nemaline, myofibrillar), congenital muscular dystrophies, metabolic myopathies (fatty acid oxidation disorders, glycogen storage disorders), and peroxisomal disorders. We excluded disorders for which the main molecular defect could not be detected reliably by NGS (repeat expansions and structural variants), such as fascioscapulohumeral dystrophy. Mitochondrial genes (mtDNA and nuclear) were excluded as well, owing to the large number of genes involved. The gene list was circulated among collaborators with special expertise in Pompe disease, medical and biochemical genetics, and neurogenetics. The final gene panel comprised 78 genes (Additional file 1: Table A). In order to design oligonucleotide probes for sequence enrichment, we delineated the genomic coordinates of exons of selected genes (all alternate transcripts) based on GRCh37/hg19 genome assembly and RefSeq gene definition (11/2013). Genomic coordinates were sent to Nimblegen Bioinformatics Production team (Madison, USA) to complete the design of the oligonucleotide probes, based on the exome enrichment kit V3. We allowed up to five perfect matches on the human genome for each probe to maximize the number of selected genomic regions covered, when necessary. In-solution hybridization oligonucleotide probe library was produced accordingly to SeqCapEZ® technology (Roche/Nimblegen)

### Determination of the analytical validity of the gene panel

We analysed 20 Pompe patients and the NA12878 CEPH cell line to determine the sensitivity and specificity of the gene panel. The genome of NA12878 has been analyzed by multiple sequencing platforms and high quality confident genotypes are available to assess sensitivity and specificity (http://genomeinabottle.org) [25]. DNA was obtained from known Pompe patients, followed at the Division of Medical Genetics at Duke University Medical Center. All patients have been diagnosed on the basis of decreased acid alpha-glucosidase enzyme activity and had results from *GAA* Sanger sequencing available. Known mutations and polymorphisms were used for comparison with results from NGS (Additional file 1: Table B).

### Next-generation sequencing

Sequencing of the gene panel by NGS was performed as follows: DNA from samples was fragmented to a mean size of ~350bp by sonication (Covaris M220). Then, sequencing libraries were prepared with TruSeq® library kit (Illumina) according to the manufacturer’s protocol, except for the following modifications at the library amplification step by LM-PCR: in order to minimize amplification bias related to GC content, we substituted the provided polymerase by the KAPA HIFI HotStart polymerase (KAPA biosystems), and we used the following amplification conditions with initial denaturation for 45 sec at 98 °C, followed by seven cycles with 15 sec at 98 °C, 30 sec at 60 °C and 30 sec at 72 °C, ending with final extension step 1 min at 72 °C. We then performed enrichment of exonic and flanking intronic sequences of selected genes in 12 samples in a multiplex reaction using our custom in-solution hybridization oligonucleotide probe library (SeqCapEZ®, Nimblegen). We followed the manufacturer’s protocol, but decreased the number of post-capture amplification cycles to 13 to minimize amplification bias related to GC content. Enrichment efficiency was measured by quantitative PCR for one GC-rich locus (*GAA* exon 14, 66 % GC content) and 2 loci with average GC-content (*TPM1* exon 1, 57 % GC; *MYBPC3* exon 6, 53 % GC). Multiplexed samples were sequenced if they met a minimal acceptable enrichment factor of 800-1000X. Sequencing was done on a MiSeq® sequencer (Illumina) using paired-end protocol (150 pb) and V2 chemistry. On average, 12.4 millions of pass filter reads were produced per sequencing run with 95.4% of reads having an average Phred score quality above Q30 (1 error in 1000 bases).

### Bioinformatics analysis

We analyzed the sequencing data on a Linux-based bioinformatics pipeline developed by MUGQIC (https://bitbucket.org/mugqic/mugqic_pipelines). Briefly, 1) raw reads were trimmed using Trimmomatic (version 0.32) [26], 2) sequence alignment was performed with Burrows-Wheeler Aligner (version 0.7.10) [27], 3) genetic variations (SNP and indels) were called with haplotypeCaller using the Genome Analysis Toolkit (version 3.2.2) [28, 29] with prior local realignment, base recalibration and removal of PCR duplicates using Picard (version 1.123, http://broadinstitute.github.io/picard/), 4) gene annotation was performed with SnpEff/SnpSift (version 3.6, including SIFT, Polyphen2, MutationTaster predictions) [30] with an additional in-house script to annotate variations present in ClinVar database [31], 5) a filtering process removed variations outside targeted sequences, with population frequency >1 % (dbSNP 138 and ExAC 0.3; http://exac.broadinstitute.org), genotype quality less than Q30 or present in 3/20 or more local controls sequenced on the same platform (therefore considered as artefacts). In order to identify single exon deletions in the *GAA* gene, we manually inspected the read coverage depth across *GAA* for each patient compared to NA12878 cell line, using the Integrative Genomics Viewer (see Additional file 1: Figure A) [32]. Small deletions (less than 3 exons), remain challenging for current software [33–35]. Coverage depth was calculated using Browser Extensible Data (BED) Tools [36] and SAMtools [37] from the Galaxy web-based platform [38], as described in detail in the Additional file 1.

### Measurement of the acid alpha-glucosidase enzyme activity in dried blood spots (DBS) by tandem mass spectrometry

The method for the acid alpha-glucosidase activity measurements in DBS was adapted from Dajnoki et al. [39] and is described in detail in the Additional file 1. The enzyme activity was analyzed in 3.2 mm diameter DBS discs. The acid alpha-glucosidase substrate ([7-benzoylamino-heptyl)-{2-[4-(3,4,5-trihydroxy-6-hydroxymethyl-tetrahydro-pyran-2-yloxy)-phenylcarbamoyl]-ethyl}-carbamic acid tert-butyl ester] and its internal standard (IS) [7-d5-benzoylamino-heptyl)-[2-(4-hydroxyphenylcarbamoyl)-ethyl]-carbamic acid tertbutyl ester] were manufactured by Genzyme, A Sanofi Division (Framingham, MA) and distributed by the Centers for Disease Control and Prevention (CDC), Newborn Screening Branch, (Atlanta, GA). The product of the enzymatic reaction and its internal standard were analyzed by tandem mass spectrometry using a Quattro Micro (Waters Corp.) in the multiple reaction monitoring mode in positive electrospray ionization.

### Assessment of clinical utility of the gene panel

**Fig. 1:**
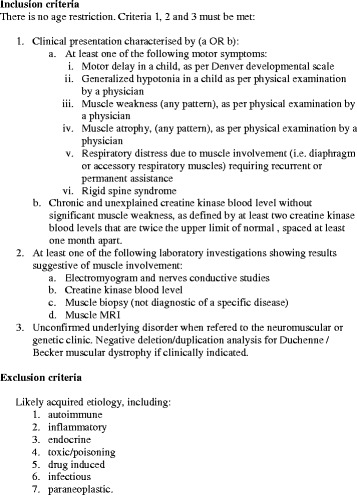
Inclusion and exclusion criteria

We recruited pediatric and adult patients with undetermined muscle disorders referred to or already followed at specialized neuromuscular or genetic clinics in different provinces in Canada (Sherbrooke, Saguenay, Montreal in Quebec and Calgary, Alberta). Inclusion and exclusion criteria are shown in Fig. 1. Each participant provided written informed consent. The study was approved by the institutional ethics committee of the Université de Sherbrooke (project 12-208). A blood sample was drawn from each participant for DNA extraction (QIAamp DNA blood mini kit, Qiagen) and a DBS specimen was collected to measure acid alpha-glucosidase enzyme activity. For 24 patients, whole blood was collected in sodium heparinized tubes and enzyme testing was performed on leucocytes by previously described standard spectrophotometric assay [40].

NGS followed by bioinformatic analysis was then performed on the DNA sample as described above. Coverage depth of exons of *GAA* in all patients was manually reviewed for heterozygous exon deletions as described in 2.4, owing to the relatively high frequency of whole exon 18 deletion. Similarly, in patients with a single causal variation in recessive disorders, we searched the relevant gene for heterozygous exon deletion. Suspected heterozygous exon deletions were confirmed by qPCR analysis or Sanger sequencing if breakpoints were known. In patients who did not have causal variations identified, 6 exons with consistently insufficient coverage (<20X; *SGCB* exon 1, *SEPN1* exon 1 and 3, *PLEC* exon 32, *FKRP* part of exon 4, *PEX10* exon 1) were sequenced to exclude mutations by the Sanger method on a 3730XL DNA analyzer (Applied Biosystems) at McGill University and Genome Quebec Innovation Center. Putative causal variations were sequenced in parents when available and were classified accordingly to the ACMG recommendations [41].

## Results

The gene panel comprises *GAA* along with 77 other genes causing muscle disorders that are considered in the differential diagnosis of LOPD [4, 24]. The complete gene list is available in the Additional file 1 section (Table A). Before investigating the clinical utility of the gene panel, we sought to determine the analytical validity of our method across all targeted genes and more specifically for *GAA*, the gene responsible for Pompe disease. We sequenced the NA12878 CEPH cell line in two independent sequencing runs, among known Pompe patients and neuromuscular patients for a total of 12 samples per sequencing run.

### Analytical validity of the gene panel

We determined the sensitivity and specificity across all selected genes by performing a comparison of observed genotypes with reference genotypes of the CEPH cell line NA12878. The genome of NA12878 has been analyzed by multiple sequencing platforms and high quality reliable genotypes are available to assess sensitivity and specificity (http://genomeinabottle.org) [25]. We observed a sensitivity of 100 % (95 % C.I.: 99–100 %) using a set of 373 known variations (359 SNV and 14 indels) across all selected genes. Median coverage was 216X and only 6/1720 exons (0.3 %) did not show a coverage depth consistently above 20X, which would need Sanger sequencing to exclude variations. All *GAA* exons were successfully covered with >20X. In order to calculate the specificity, we restricted our analysis to variations located in exons and splice site junctions (+/- 5 bp), which are more relevant for disease causing variations. Deep intronic regions contain more frequently repetitive sequences that yield higher false positive rates. A total of 208 variations (204 SNV and 4 indels) were detected in exons and splice site junctions of selected genes. Among these, four variations (1 indel) were not found in the reference genotypes, which yielded a specificity of 98 % (95 % C.I.: 95–99 %).

We analysed the sequencing data of 20 Pompe patients with known mutations and polymorphisms to determine more specifically the sensitivity of our method for variations located in the *GAA* gene. Our gene panel showed a 100 % sensitivity (95 % C.I.: 98–100 %) based on the analysis of 197 known single nucleotide variations and indels (36 SNV and 6 indels; Additional file 1: Table B). Median coverage of *GAA* for each Pompe patient sequenced ranged from 72X to 150X. All *GAA* exons were successfully covered with >20X. In addition, deletion of whole exon 18 was correctly detected by manual read depth inspection in 5/5 Pompe patients.

### Validation of DBS acid alpha-glucosidase activity by tandem mass spectrometry

For the tandem mass spectrometry method validation, quality control (QC) DBS at four different enzyme levels of activity (from low QC1 to high QC4) were supplied by the CDC and used to measure the intra- and interday assay precision of the method (Table 1). Fifteen DBS from diagnosed Pompe patients were analyzed as positive quality controls and 49 DBS from healthy controls were analyzed to establish normal values for the GAA enzyme activity. The GAA activity was abnormal, with mean 0.51 (observed range nd-3.09) μmol/h/L for the 15 diagnosed Pompe patients and normal, with mean 8.57 (observed range 4.72–16.36) μmol/h/L for the 49 healthy controls. These results show that our method, which is scaled down from the current large-scale protocol [39] and is thus less expansive, provides a similar high quality of results.

**Table 1:** GAA enzyme activity intra- and interday assays using DBS from low to high quality controls (QCs)

	Intraday assays (n = 5)			
	QC1	QC2	QC3	QC4
Mean (μmol/h/L)	0.28	0.84	6.79	13.67
Range (μmol/h/L)	0.20–0.49	0.75–0.89	6.20–7.64	11.50–15.83
RSD%	41.42	6.44	8.70	12.79
	Interday assays (n = 5)			
	QC1	QC2	QC3	QC4
Mean (μmol/h/L)	0.33	1.00	7.29	13.70
Range (μmol/h/L)	(0.17–0.53)	(0.69–1.44)	(6.85–7.69)	(12.08–15.09)
RSD%	38.75	27.17	4.22	9.37

*RSD* relative standard deviation

### Clinical utility of the gene panel

We assessed the clinical utility of the gene panel in a cohort of pediatric and adult patients referred and followed at specialized neuromuscular and genetic clinics for an undetermined suspected muscle disease. A total of seven pediatric and 27 adult patients were recruited and sequenced. Median coverage for each patient ranged from 81X to 361X, for a mean of 168X. Most patients presented with proximal muscle weakness or a more diffuse pattern with distal involvement (Table 2). At the time of recruitment 24/34 patients (71 %) had undergone a muscle biopsy and 15/34 (44 %) had at least one single gene test performed, but remained without a diagnosis.

**Table 2:** Recruited patients with undermined muscle disease

Clinical presentation	Nbr of patients (pediatric patients)	Proportion of clinical presentations (%)	Proportion of diagnoses (%)
Motor delay or generalized hypotonia	4 (4)	4/34 (11.8%)	2/11 (18%)
Limb-girdle muscle weakness	17 (1)	17/34 (50.0%)	6/11 (55%)
Proximal and distal limb muscle weakness	4 (2)	4/34 (11.8%)	3/11 (27%)
Other muscle weakness	3 (0)	3/34 (8.8%)	0/11 (0%)
Chronic and unexplained hyperCKemia	6 (0)	6/34 (17.6%)	0/11 (0%)
Total	34 (7)	100%	11/34 (32%)

We identified one Pompe patient harbouring 2 known pathogenic variations (P29), the c.-32-13T > G mutation, associated with LOPD, and the recurrent exon 18 deletion. In four other patients, we identified pathogenic variations supportive of an inherited muscle disorder (Table 3), for a diagnostic yield of 15 %. Likely pathogenic variations, variants of uncertain clinical significance or a combination of both were found in six patients, suggesting additional diagnoses. In three of these patients, revision of available muscle biopsy or additional immunohistochemistry supported the diagnosis (P22, P25, P27). In the three others, family history was in agreement with expected heritability, but samples were not available for segregation study. In total, a putative diagnosis was found in 11/34 (32 %) patients (Table 3). Most diagnoses showed various types of limb-girdle muscular dystrophies, although some had atypical presentations with respect to the causative gene (P25, P28, P29; see section 3.4). Acid alpha-glucosidase enzyme activity yielded normal results in all but one patient (P29), while abnormally low enzyme activity for P29 supported the diagnosis of Pompe disease made by molecular analysis (Table 3). One DBS sample was rejected on the basis of low control enzymes in addition to acid alpha-glucosidase, likely secondary to insufficient biological material (patient P27). Enzyme testing could not be repeated on a second sample, but a putative causal variation in the *VCP* gene was found. Delay from initial symptoms to diagnosis ranged from 1 year in pediatric patients to more than 20 years in adults. In patients without diagnosis, we observed 0–5 heterozygous variants of uncertain significance altering the protein sequence of different recessive disorders.

**Table 3:** Putative causal variations in patients with undetermined neuromuscular diseases

Patient	Age (years)	Age of onset (years)	Clinical presentation	Gene	Variation 1 (interpretation)	Variation 2 (interpretation)	Disorder inheritance
P11	10	6	Proximal and distal limb muscle weakness	*COL6A1*	c.1059 + 1 G > A^a^(Pathogenic)	-	Bethlem myopathy (AD)
P13	11	0.3	Motor delay	*DNAJB6*	c.525C > G p.Phe175Leu (VUS)	-	LGMD1E (AD)
P17	21	11	Proximal and distal limb muscle weakness	*FKTN*	c.607C > T p.Arg203Ter (Likely path.)	c.1238G > T p.Cys413Phe (VUS)	LGMD2M (AR)
P20	35	25	Limb-girdle muscle weakness	*ANO5*	c.1295C > G p.Ala432Gly (Pathogenic)	c.989dupT p.Leu 330Phefs (Pathogenic)	LGMD2L (AR)
P21	56	N/A	Limb-girdle muscle weakness	*COL6A3*	c.5658G > A p.Arg1886Cys (VUS)	-	Bethlem myopathy (AD)
P22	46	<26	Limb-girdle muscle weakness	*DYSF*	c.2448 + 2 T > G (Likely path.)	c.3351_3352delAG p.Thr1117Thrfs (Likely path.)	LGMD2B (AR)
P25	48	N/A	Proximal and distal limb muscle weakness	*NEB*	c.24339_24342del p.Leu8113Leufs (Likely path.)	c.24113C > A p.Ser8038Ter (Likely path.)	Nemaline myopathy (AR)
P27	60	57	Limb-girdle muscle weakness	*VCP*	c.1158T > C p.Lys386Glu (VUS)	-	Inclusion body myopathy (AD)
P28	43	<10	Limb-girdle muscle weakness (female)	*DMD*	c.7683G > A (p.Trp2561Ter) (Pathogenic)	-	Duchenne muscular dystrophy (XL)
P29	48	<36	Limb-girdle muscle weakness	*GAA*	c.-32-13T > G (Pathogenic)	Exon 18 deletion (Pathogenic)	Pompe disease (AR)
P30	1	0	Motor delay and generalized hypotonia	*POMT2*	c.1997A > G^b^ p.Tyr666Cys (Pathogenic)	c.1997A > G^b^ p.Tyr666Cys (Pathogenic)	Muscular dystrophy-dystroglycanopathy (AR)

^a^de novo variation; ^b^Parental heterozygous carrier status confirmed; *N/A* Not available, *VUS* variant of uncertain significance. Variations were classified accordingly to the ACMG recommendations

### Atypical cases

Patient P25 showed unusual late onset of symptoms for a patient with *NEB* mutations. The patient had normal birth history and psychomotor development. Objective diffuse mild muscle weakness was only documented in adulthood. However, muscle weakness might have preceded adulthood since reduced body weight was reported in early teenage years with decreased tolerance for moderate intensity exercise. However, no further investigation was performed at that time, until adulthood. Muscle biopsy showed nemaline inclusions in a configuration supporting a diagnosis of CAP myopathy. Sequencing of *TPM2*, *TPM3* and *ACTA1* was negative. The patient remains fully ambulatory. Patient 28 showed an unusual severe and early onset of symptoms for a heterozygous carrier of a truncating mutation in *DMD*. She presented with limb girdle weakness before 10 years of age, high CK and limited long distance walking. There was no family history of affected males with muscular dystrophy, and she had a daughter who showed similar symptoms at 11 years. Muscle biopsy performed in the context of autosomal dominant limb-girdle dystrophy failed to support a specific diagnosis. Patient P29, who was diagnosed with Pompe disease, showed a peculiar phenotype, which likely contributed to delay in diagnosis. The patient is a female born prematurely at 32 weeks of gestational age. She showed motor and language delay, with walking at 3 years and first words around 2.5 years. Neuropsychological evaluation revealed a mild intellectual deficit. She received a diagnosis of cerebral palsy early in the course. She was otherwise known for obesity, hypertension and past history of seizures. Although she was reported to present long lasting gait disturbances, it was only at the age of 36 years that progression of the disease made her unable to climb stairs at home and she began to use a walker. Weakness progressed and she became wheelchair-bound at the age of 47 years. At 48 years old, respiratory insufficiency was noted and treated with non-invasive ventilation at night. A muscle biopsy was performed and showed glycogen accumulation, and the patient was referred to the genetics clinic for suspicion of Pompe disease. Diagnosis was established on the basis of the acid alpha-glucosidase activity and mutation analysis as described above. In short, the delay from initial symptoms to diagnosis was at least 12 years and possibly more, owing to the long lasting history of gait disturbances. Enzyme replacement therapy was initiated in the weeks following the diagnosis and response is currently being assessed.

## Discussion

Our work highlights the high clinical utility of gene panels in patients with suspected muscle disorders and their potential to facilitate the diagnosis of patients showing non-specific muscle weakness or atypical phenotypes. This gene panel approach might further increase diagnosis of muscle conditions and reduce diagnostic delay. We propose that gene panels should be used as a first-tier test in patients with suspected muscle disorders of undetermined etiology. With decreasing sequencing costs and increasing availability of NGS-based tests, there are several advantages of performing gene panels early in the investigation of patients with muscle weakness.

Firstly, gene panels offer a higher clinical utility than any single disorder test by enabling screening for several diseases with overlapping phenotypes at the same time. This is especially useful in the context of patients with non-specific muscle patterns, such as children with motor delay or adults with limb-girdle patterns which are frequently encountered in neuromuscular and genetics clinics. There are more than 25 different types of limb-girdle muscular dystrophies, which often could not be distinguished clinically [42]. Moreover, muscle biopsy and immunostains are of very limited utility to target molecular tests in some instances, such as *ANO5* (patient P25) [42]. In these latter cases, diagnosis could require several molecular single gene tests which can be prohibitive when considering the broader differential diagnosis of limb-girdle muscle weakness: Pompe disease, dystrophinopathies, Bethlem myopathy, myofibrillar myopathies, metabolic myopathies and others [42]. All these conditions would be readily diagnosed with a gene panel like the one we have developed. In the present study, the rate of diagnosis reached up to 32 % of patients tested, with 50 % presenting with limb-girdle weakness. Since our cohort is enriched for patients who have been followed for years in specialized clinics without diagnosis and 44 % of patients had previous negative molecular tests results, one would expect a higher rate of diagnosis if the gene panel was performed as a first-tier test. Previous studies performing targeted next generation sequencing, similarly as a second tier test in patients with muscular dystrophies, reported diagnostic yields ranging from 16 to 65 % [43–45]. Even if no treatment is available for the majority of these conditions, benefits of genetic diagnosis include more accurate prognostic information, genetic counselling, family screening and prenatal diagnosis in severe cases.

Secondly, use of gene panels in patients with muscle weakness could facilitate the diagnosis of atypical phenotypes, and reduce diagnostic delay. Delay in diagnosis varied from 1 year in pediatric patients to more than 20 years in adults included in the present study. Patients P25, P28, and P29 showed atypical or unusual characteristics that likely contributed to delayed diagnosis. P25 showed unusual late onset of symptoms for *NEB* mutations and biopsy showed features supporting a diagnosis of CAP myopathy. Most cases of *NEB* mutations present in the neonatal period or within the first years of life, although some late-onset presentations have been described [46–48]. Moreover, mutations in *NEB* was not a known cause of CAP myopathy at the time of clinical evaluation and had only been reported recently in a single case [49]. Patient P28, harbouring a truncating mutation in *DMD,* presented unusual severe limb-girdle weakness of pediatric onset. In the two largest reported series of *DMD* mutation carriers, the proportion of manifesting carriers varied from 5 to 22 % and the pediatric cases remained rare [50, 51]. However, in recent studies, female carriers with pediatric onset of muscle weakness are becoming increasingly recognized [52, 53]. In our case, absence of affected male and a similarly affected daughter on family history further contributed to confusion about inheritance, leading to an erroneous working diagnosis of autosomal limb girdle dystrophy. Finally, the peculiar disease course and spectrum of clinical manifestations of P29, possibly related in part to premature birth and complications, certainly contributed to delay in the recognition of Pompe disease by at least 12 years. This is not unusual as final diagnosis was reported to be made almost 10 years after the onset of symptoms in LOPD [15]. Although this is debatable, earlier accessibility to our gene panel at the age of 36 years while progression to limb-girdle weakness became significant could have led to a more rapid diagnosis for this patient, and possibly a different outcome at 48 years of age. While we agree that Pompe disease should be considered in all patients with limb-girdle weakness, in practice it is likely that screening through acid alpha-glucosidase activity will not necessarily be requested by every adult neurologist owing in part to the low frequency of LOPD. In our view, gene panels represent a more comprehensive first-tier diagnostic option in this type of scenario, and are more likely to be adopted by the medical community. Nevertheless, it remains to be determined whether broad adoption of gene panel as a first-tier test would result in significant reduced delay in the diagnosis of LOPD leading to an impact on treatment outcome. This question is timely as next generation sequencing becomes more widespread in al practice clinic and also considering the current populations of children and adult who will not benefit from ongoing implementation of newborn screening.

The pitfalls of using gene panels as first-tier analysis in the place of enzyme activity for identification of Pompe disease include the identification of variants of unknown significance (VUS), detection of a single heterozygous mutation, and limited performance of current bioinformatic tools to detect deletions and duplications from targeted NGS data [33–35, 41]. The VUS rates will greatly vary from gene to gene, but large collaborative efforts to share mutation data, such as ClinVar, are underway to minimise this limitation [31]. In Pompe disease, 19 % of 84 *GAA* variants reported in ClinVar that were not classified as likely benign, are of uncertain clinical significance (June 2015). The Pompe disease mutation database (www.pompecenter.nl/, June 2015) lists 71 variations with unknown effect among 426 variations (17 %) that are not classified as likely benign [54]. Consequently, most patients present with two pathogenic alleles, but enzyme testing may be needed as reflex testing in those harbouring a VUS [55–58]. In the present study, we did not identify VUSs in *GAA* in our cohort of 34 patients, although we observed 0–5 VUS per patient across all genes analyzed. Pompe patients with a single heterozygous mutation identified are uncommon and represent 2–17 % of patients with enzyme-based diagnosis from several case series [55–58]. Assessment of recurrent exon 18 deletion and other deletions in the *GAA* gene is critical to minimize this number. Currently, bioinformatic identification of deletions and duplications with targeted NGS-based data is of limited sensitivity, especially for less than three exons [33–35]. However, we managed to detect the recurrent exon 18 deletion from manual inspection of read coverage compared to a control. As for VUSs, reflex enzyme testing is necessary here to exclude that a *GAA* mutation was missed. Owing to these limitations, testing for acid alpha-glucosidase activity would appear the test of choice in the context of a strong clinical suspicion of Pompe disease.

The current state of NGS technology favors the use of gene panels over exome sequencing in the clinical setting, although this is likely to change in upcoming years. Beside the higher cost of exome sequencing, the main disadvantage is related to more incomplete coverage of exonic sequences (~5 %) owing to generally lower average coverage compared to panels [59–61]. Raising the average coverage of exome sequencing to reach those of gene panels will only lead to further increase of its cost disadvantage. In addition, it has to be demonstrated that the diagnostic yield of exome sequencing is significantly superior to a well-designed clinical panel [59, 60]. Variants detected in genes that are ambiguously or not yet related to the patient’s phenotype are likely to be of limited clinical utility. Interestingly, a recent study using exome sequencing showed similar yield compare to our study with 22 of 74 patients (30 %) classified as muscular dystrophies and related disorders [62]. Another study, from the Emory Genetics Laboratory, reported that exome sequencing missed about 18 % of causative pathogenic variants detected by their neuromuscular gene panel [60]. Obviously, exome sequencing raises additional issues including increased number of VUSs, incidental findings and higher laboratory infrastructure cost for clinical implementation.

## Conclusion

Our work highlights the high clinical utility of gene panels in patients with muscle disorders and its potential to facilitate the diagnosis of patients showing non-specific muscle weakness or atypical phenotypes. This could potentially lead to earlier diagnosis, if performed as a first-tier test. It remains to be determined if this would have a significant impact on treatment outcome for LOPD.

## Additional file

Additional file 1:
**Supplemental information.** (DOCX 157 kb)

## Abbreviations

DBS: dried blood spot
ERT: enzyme replacement therapy
LOPD: late-onset Pompe disease
NGS: next generation sequencing
VUS: variant of unknown significance
