# Supplementary material for: Diagnosis of late-onset Pompe disease and other muscle disorders by next-generation sequencing
Source: Orphanet J Rare Dis. 2016 Jan 25;11:8. doi: 10.1186/s13023-016-0390-6 (PMC4727295; doi:10.1186/s13023-016-0390-6)
Supplement: Additional file 1: — Supplemental information. (DOCX 157 kb) [file 13023_2016_390_MOESM1_ESM.docx]

**Additional file 1: Supplemental information**

| **Table A: Selected genes included in the gene panel** | |  |
| --- | --- | --- |
| Gene Symbols | Diseases |  |
|  |  |  |
| Neuromuscular disorders (61 genes) | |  |
|  |  |  |
| *ACTA1* | Myopathy, nemaline, 3 |  |
| *AGL* | Glycogen storage disease III |  |
| *ANO5* | Muscular dystrophy, limb-girdle, type 2L |  |
| *BAG3* | Myopathy, myofibrillar, BAG3-related |  |
| *CAPN3* | Muscular dystrophy, limb-girdle, type 2A |  |
| *CAV3* | Rippling muscle disease ; Muscular dystrophy, limb-girdle, type IC |  |
| *CFL2* | Nemaline myopathy 7 |  |
| *CHRNB1* | Myasthenic syndrome, congenital, associated with acetylcholine receptor deficiency |  |
| *CHRNE* | Myasthenic syndrome, congenital, associated with acetylcholine receptor deficiency |  |
| *COL6A1* | Bethlem myopathy ; Ullrich congenital muscular dystrophy |  |
| *COL6A2* | Bethlem myopathy; Ullrich congenital muscular dystrophy |  |
| *COL6A3* | Bethlem myopathy; Ullrich congenital muscular dystrophy |  |
| *CPT2* | Myopathy due to CPT II deficiency |  |
| *DES* | Scapuloperoneal syndrome, neurogenic, Kaeser type ; Myopathy, myofibrillar, 1 |  |
| *DMD* | Becker muscular dystrophy ; Duchenne muscular dystrophy |  |
| *DNAJB6* | Muscular dystrophy, limb-girdle, type 1E |  |
| *DYSF* | Muscular dystrophy, limb-girdle, type 2B |  |
| *EMD* | Emery-Dreifuss muscular dystrophy 1, X-linked |  |
| *ETFA* | Multiple acyl-CoA dehydrogenase deficiency; MADD |  |
| *ETFB* | Multiple acyl-CoA dehydrogenase deficiency; MADD |  |
| *ETFDH* | Multiple acyl-CoA dehydrogenase deficiency; MADD |  |
| *FHL1* | Scapuloperoneal myopathy, X-linked dominant |  |
|  | Emery-Dreifuss muscular dystrophy 6, X-linked (Myopathy, X-linked, with postural muscle atrophy) | |
|  | Myopathy, reducing body, X-linked, severe early-onset |  |
|  | Myopathy, reducing body, X-linked, childhood-onset |  |
| *FKRP* | Muscular dystrophy-dystroglycanopathy (congenital with or without mental retardation), type B, 5 | |
|  | Muscular dystrophy-dystroglycanopathy (limb-girdle), type C, 5 |  |
|  | Muscular dystrophy-dystroglycanopathy (congenital with brain and eye anomalies), type A, 5 | |
| *FKTN* | Muscular dystrophy-dystroglycanopathy (congenital with brain and eye anomalies), type A, 4 | |
|  | Muscular dystrophy-dystroglycanopathy (limb-girdle), type C, 4 |  |
|  | Muscular dystrophy-dystroglycanopathy (congenital w/o mental retardation), type B, 4 |  |
| ***GAA*** | **Glycogen storage disease II (pompe disease)** |  |
| *GBE1* | Glycogen storage disease IV |  |
| *HADHA* | Long-chain hydroxy acyl-coA deshydrogenase | |
| *ISPD* | Muscular dystrophy-dystroglycanopathy (congenital with brain and eye anomalies), type A, 7 | |
| *KBTBD13* | Nemaline myopathy 6 |  |
| *LAMA2* | Muscular dystrophy, congenital merosin-deficient |  |
| *LAMP2* | Danon disease |  |
| *LARGE* | Muscular dystrophy-dystroglycanopathy (congenital with mental retardation), type B, 6 |  |
|  | Muscular dystrophy-dystroglycanopathy (congenital with brain and eye anomalies), type A, 6 | |
|  |  |  |
| *LMNA* | Muscular dystrophy, limb-girdle, type 1B ; Emery-Dreifuss muscular dystrophy 2, AD |  |
|  | Muscular dystrophy, congenital |  |
| *MUSK* | Myasthenic syndrome, congenital, associated with acetylcholine receptor deficiency |  |
| *MYH7* | Scapuloperoneal syndrome, myopathic type ; Myopathy, myosin storage |  |
| *MYOT* | Muscular dystrophy, limb-girdle, type 1A |  |
| *NEB* | Nemaline myopathy 2, autosomal recessive |  |
| *PFKM* | Glycogen storage disease VII |  |
| *PLEC* | Muscular dystrophy, limb-girdle, type 2Q |  |
| *PLEKHG5* | Spinal muscular atrophy, distal, autosomal recessive, 4 |  |
| *POMGNT1* | Muscular dystrophy-dystroglycanopathy (congenital with brain and eye anomalies), type A, 3 | |
|  | Muscular dystrophy-dystroglycanopathy (congenital with mental retardation), type B, 3 |  |
|  | Muscular dystrophy-dystroglycanopathy (limb-girdle), type C, 3 |  |
| *POMT1* | Muscular dystrophy-dystroglycanopathy (congenital with brain and eye anomalies), type A, 1 | |
|  | Muscular dystrophy-dystroglycanopathy (limb-girdle), type C, 1 |  |
|  | Muscular dystrophy-dystroglycanopathy (congenital with mental retardation), type B, 1 |  |
| *POMT2* | Muscular dystrophy-dystroglycanopathy (congenital with brain and eye anomalies), type A, 2 | |
|  | Muscular dystrophy-dystroglycanopathy (congenital with mental retardation), type B, 2 |  |
|  | Muscular dystrophy-dystroglycanopathy (limb-girdle), type C, 2 |  |
| *PYGM* | McArdle disease |  |
| *RAPSN* | Myasthenic syndrome, congenital, associated with acetylcholine receptor deficiency |  |
| *SEPN1* | Myopathy, congenital, with fiber-type disproportion ; Muscular dystrophy, rigid spine, 1 |  |
| *SGCA* | Muscular dystrophy, limb-girdle, type 2D |  |
| *SGCB* | Muscular dystrophy, limb-girdle, type 2E |  |
| *SGCD* | Muscular dystrophy, limb-girdle, type 2F |  |
| *SGCG* | Muscular dystrophy, limb-girdle, type 2C |  |
| *SLC22A5* | Carnitine deficiency, systemic primary |  |
| *SLC25A20* | Carnitine-acylcarnitine translocase deficiency |  |
| *TCAP* | Muscular dystrophy, limb-girdle, type 2G |  |
| *TNNT1* | Nemaline myopathy, Amish type |  |
| *TPM2* | Nemaline myopathy |  |
| *TPM3* | Nemaline myopathy 1, autosomal dominant |  |
| *TRIM32* | Muscular dystrophy, limb-girdle, type 2H |  |
| *TRPV4* | Scapuloperoneal spinal muscular atrophy |  |
| *TTN* | Myopathy, proximal, with early respiratory muscle involvement |  |
|  | Muscular dystrophy, limb-girdle, type 2J |  |
| *VCP* | Inclusion body myopathy with early-onset Paget disease and frontotemporal dementia |  |
| *VLCAD* | Very long-chain acylcoA deshydrogenase |  |
| Peroxisomal disorders (17 genes) | |  |
|  |  |  |
| *ACOX1* | Peroxisomal acyl-CoA oxidase deficiency; Zellweger spectrum |  |
| *HSD17B4* | D-bifunctional protein deficiency; Zellweger spectrum |  |
| *PEX1* | Peroxisome biogenesis disorder; Zellweger spectrum |  |
| *PEX2* | Peroxisome biogenesis disorder; Zellweger spectrum |  |
| *PEX3* | Peroxisome biogenesis disorder; Zellweger spectrum |  |
| *PEX5* | Peroxisome biogenesis disorder; Zellweger spectrum |  |
| *PEX6* | Peroxisome biogenesis disorder; Zellweger spectrum |  |
| *PEX10* | Peroxisome biogenesis disorder; Zellweger spectrum |  |
| *PEX11A* | Peroxisome biogenesis disorder; Zellweger spectrum |  |
| *PEX11B* | Peroxisome biogenesis disorder; Zellweger spectrum |  |
| *PEX11G* | Peroxisome biogenesis disorder; Zellweger spectrum |  |
| *PEX12* | Peroxisome biogenesis disorder; Zellweger spectrum |  |
| *PEX13* | Peroxisome biogenesis disorder; Zellweger spectrum |  |
| *PEX14* | Peroxisome biogenesis disorder; Zellweger spectrum |  |
| *PEX16* | Peroxisome biogenesis disorder; Zellweger spectrum |  |
| *PEX19* | Peroxisome biogenesis disorder; Zellweger spectrum |  |
| *PEX26* | Peroxisome biogenesis disorder; Zellweger spectrum |  |
|  |  |  |
|  |  |  |

**Table B. Forty-two unique known mutations and polymorphisms in 20 Pompe patients determined by Sanger sequencing**

|  |  |  |
| --- | --- | --- |
| Gene symbol | Variation | Type |
| GAA | c.-32-17_-32-10delinsTCCCTGCTGAGCCTCCTACAGGCCTCCCGC | Indel |
| GAA | c.-32-13T>C | SNV |
| GAA | c.324T>C | SNV |
| GAA | c.525delT | Indel |
| GAA | c.547-4C>G | SNV |
| GAA | c.596A>G | SNV |
| GAA | c.642C>T | SNV |
| GAA | c.668G>A | SNV |
| GAA | c.743T>C | SNV |
| GAA | c.858+7_858+8insAGCGGGC | Indel |
| GAA | c.876C>T | SNV |
| GAA | c.921A>T | SNV |
| GAA | c.955+12G>A | SNV |
| GAA | c.1143delC | Indel |
| GAA | c.1203G>A | SNV |
| GAA | c.1327-18A>G | SNV |
| GAA | c.1374C>T | SNV |
| GAA | c.1438-19G>C | SNV |
| GAA | c.1445C>G | SNV |
| GAA | c.1447G>A | SNV |
| GAA | c.1477C>T | SNV |
| GAA | c.1548G>A | SNV |
| GAA | c.1551+49C>A | SNV |
| GAA | c.1581G>A | SNV |
| GAA | c.1642G>T | SNV |
| GAA | c.1726G>A | SNV |
| GAA | c.1827delC | Indel |
| GAA | c.1880C>T | SNV |
| GAA | c.1888+21G>A | SNV |
| GAA | c.1978C>T | SNV |
| GAA | c.2040+20A>G | SNV |
| GAA | c.2065G>A | SNV |
| GAA | c.2133A>G | SNV |
| GAA | c.2221G>A | SNV |
| GAA | c.2238G>A | SNV |
| GAA | c.2331+20G>A | SNV |
| GAA | c.2331+24T>C | SNV |
| GAA | c.2338G>A | SNV |
| GAA | c.2446G>A | SNV |
| GAA | c.2553G>A | SNV |
| GAA | c.2647-20T>G | SNV |
| GAA | c.2706delG | Indel |

SNV: single nucleotide variation

**Figure A. Sequence coverage of the last exons of *GAA* showing exon 18 deletion**


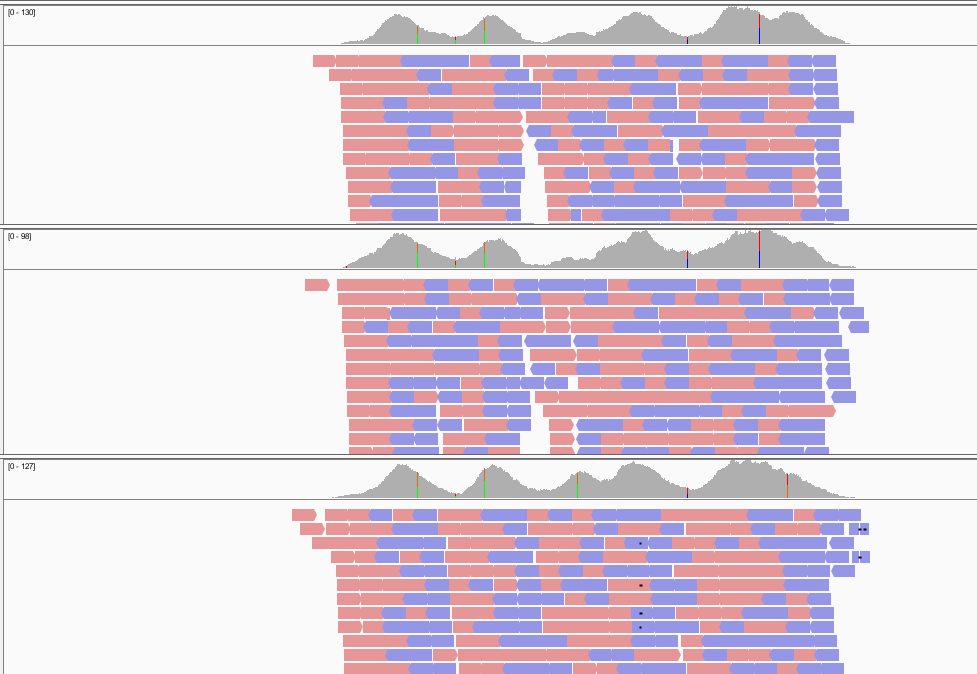

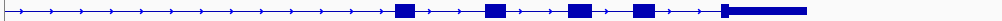


Exon 18

Deletion control

Deletion control

Number of reads

(0-130)

Number of reads

(0-98)

Number of reads

(0-127)

Normal

control

**Materials and Methods**

**Coverage depth analysis**

We calculated coverage depth of targeted sequences using Galaxy web-based platform[1]. For each sample, we used Browser Extensible Data (BED)Tools[2] to intersect aligned sequences (BAM files) with genomic coordinates of targeted sequences (BED format). We considered on target sequence reads intersecting with at least one base. Those reads were kept for the following steps. Mpileup command from SAMtools[3] was then used to generate calculate the coverage at each base position, and then we executed Filter pileup command to obtain all genomic intervals with coverage above 20X. Operate on genomics intervals/coverage command was used to calculate what proportion of targeted sequences (BED format) was covered by the output of the Filter pileup command. Genomics intervals showing coverage lower 20X were obtained by simple filter command. Standard coverage statistics were calculated using statistics/count command on the Mpileup output.

**Measurement of the acid alpha-glucosidase enzyme activity in dried blood spots (DBS) by tandem mass spectrometry**

**Reagents**

The GAA substrate ([7-benzoylamino-heptyl)-{2-[4-(3,4,5-trihydroxy-6-hydroxymethyl-tetrahydro-pyran-2-yloxy)-phenylcarbamoyl]-ethyl}-carbamic acid tert-butyl ester] and its internal standard (IS) [7-d5-benzoylamino-heptyl)-[2-(4-hydroxyphenylcarbamoyl)-ethyl]-carbamic acid tertbutyl ester] were manufactured by Genzyme, A Sanofi Division (Framingham, MA) and distributed by the Centers for Disease Control and Prevention (CDC), Newborn Screening Branch, (Atlanta, GA). ReagentPlus^®^ grade sodium phosphate monobasic (≥ 99.0%), BioUltra grade sodium citrate tribasic dihydrate (≥ 99.0%), and BioReagent grade CHAPS (3-[(3-Cholamidopropyl)dimethylammonio]-1-propanesulfonate) hydrate were purchased from Sigma-Aldrich (St-Louis, MO). Reagent grade ethyl acetate (EA) was obtained from ACP Chemicals (Montreal, Canada). Sodium hydroxide solution (1N), and Optima grade water were supplied by Fischer Scientific (Fair Lawn, NJ). Reagent grade concentrated hydrochloric acid was from Anachemia (Montreal, Canada). Acarbose (O-4,6-Dideoxy-4-[[(1S,4R,5S,6S)-4,5,6-trihydroxy-3-(hydroxymethyl)-2-cyclohexen-1-yl]amino]-α-D-glucopyranosyl-(1→4)-O-α-D-glucopyranosyl-(1→4)-D-glucose) was purchased from Toronto Research Chemicals (Toronto, Canada). Formic acid (FA) (99+%) was purchased from Acros Organics (Morris Plains, NJ). LC-MS grade methanol (MeOH) and acetonitrile (ACN) were from EMD Chemicals Inc. (Darmstadt, Germany).

**Sample collection**

Dried blood spots (DBS) were collected from 10 suspected Pompe patients and 49 healthy controls using 2.4 mm depth Sterilance^®^ lancets (Alpharetta, GA) and Whatman 903 filter papers (GE Healthcare, Little Chalfont, United Kingdom). After collection, samples were dried at room temperature for two hours and stored at -20^o^C in hermetic plastic bags containing desiccant silica gel packets.

For the method validation, quality control (QC) DBS at 4 different enzyme activity levels (from low QC1 to high QC4) were supplied by the CDC. Fifteen DBS from diagnosed Pompe patients were analyzed as positive quality controls.

**Sample preparation**

The sample preparation method was adapted from Dajnoki *et al*[4]. Briefly, 3.2 mm discs were obtained from each DBS sample (n = 1) and from a blank filter (n = 5) using an Acme Ruler (Mount Forest, Canada) punch, and deposited in 2 mL microcentrifuge tubes (Sorenson Bioscience, Salt Lake City, UT). Seventy microliters (70 µL) of the extraction buffer (20 mM Sodium phosphate, pH 7.1) were added to each tube. The samples were afterwards incubated 60 min at 37^o^C and 300 RPM in a VWR Incubating Orbital Shaker (Radnor, PA). A volume of 10 µL of each plasma or blank extract was transferred in a new tube and mixed with 15 µL of the GAA assay cocktail. This reagent was obtained by adding 1.80 mL of detergent (100 g/L CHAPS in water), 15.9 mL of buffer (0.34 M sodium phosphate monobasic + 0.17 M sodium citrate tribasic dihydrate, pH 4.0), and 0.3 mL of inhibitor (acarbose 0.8 mM in water) to a vial containing 7.91 mg of GAA substrate and 0.06 mg of IS. Each plasma or blank sample was then incubated 20 hours at 37^o^C and 225 RPM. The enzymatic reaction was quenched with 500 µL of 1:1 EA:MeOH. For liquid-liquid extraction, 400 µL of water was added to each tube and 300 µL of the upper organic phase from each sample was transferred to a new tube. Samples were evaporated to dryness under a nitrogen stream. All samples were resuspended in 200 µL of 19:1 EA:MeOH and loaded on Sep-Pak silica (1cc 100 mg) (Waters Corp, Manchester, UK) solid phase extraction cartridges preconditioned with 500 µL of 19:1 EA:MeOH. The analyzed compounds are not retained on the cartridge and the sample collection must start with the loading. The elution of the analytes was completed by adding 800 µL of 19:1 EA:MeOH twice. Finally, the eluate was evaporated to dryness and resuspended in 200 µL of the mobile phase (80% ACN/ 0.2% FA) for analysis.

For the method validation, intraday (n = 5) and interday (n = 5) analyses were performed on the 4 QCs supplied by the CDC.

**Liquid chromatography/tandem mass spectrometry analysis**

An Alliance 2795HT high performance liquid chromatography (HPLC) system (Waters Corp., Milford, MA) was used for sample injection. A junction was used instead of a chromatographic column and all the analytes were analyzed in the dead volume. The mobile phase was 80% ACN/ 0.2% FA, the flow rate 0.25 mL/ min and the injection volume (20 µL). The UPLC system was linked to a Quattro Micro (Waters) tandem mass spectrometer operated in multiple reaction monitoring (MRM) mode. Using this mode, the molecular ion of the analyte (precursor ion) is isolated in the first quadrupole, fragmented in the collision cell, and one of its specific fragments (fragment ion) is isolated in the last quadrupole for detection. The fragmentation was performed by collision-induced dissociation (CID) using argon as the collision gas. Two MRM reactions corresponding to the reaction product (*m/z* 498.30 → 398.24) and to the internal standard (*m/z* 503.33 → 403.27) were alternately recorded in centroid mode with an inter-scan delay of 0.03 s. Table C presents the parameters used for the mass spectrometer. For quantification, a 7-point calibration curve supplied by the CDC, with product/internal standard (P/IS) ratios of 0; 0.05; 0.1; 0.5; 1.0; 2.0; and 5.0 was used.

**Table C**. Mass spectrometry parameters for the analysis of the GAA reaction product and the internal standard.

**Data analysis**

The ratios of the reaction product area with the IS area for the samples, QCs and blanks were obtained using the QuanLynx version 4.1 method editor (Waters). The calibration curve was linear with the origin excluded and 1/x as weighting function. The GAA enzyme activity was calculated using Equation 1:

**Equation 1:**

**GAA activity (µmol/h/L) = 1000*(P/IS)*A/RF/T/V**

P/IS = Area of product (P)/area of internal standard (IS)

A = Amount of IS = 0.1 nmol

RF = Slope of the calibration curve

T = Incubation time = 20 h

V = Blood volume = 3.1 µL/7 (assuming that a 3.2 mm punch corresponds to 3.1 µL of blood).

All enzyme activity is subtracted by the average activity of blank filter papers (n = 3) prepared the same day.

**Additional comment on the DBS method for GAA activity**

Current DBS methods for the analysis of acid α-glucosidase (GAA) activity were developed for large-scale high-risk screening and require sophisticated robotics for sample preparation in 96-well plates[4]. We have adapted the current method to allow the sample preparation of small sample batches (0-50 samples) with less expensive benchtop instruments (orbital shaker incubator, centrifuge, pipets …) and consumables (Eppendorf tubes) commonly found in mass spectrometry laboratories. Using the Centres for Disease Control (CDC) DBS standards, 15 DBS from diagnosed Pompe patients and 49 DBS from healthy controls have proven that our less expensive scale-down method also provides high quality results similar to the current large-scale protocol.

**References**

1. Blankenberg D, Von Kuster G, Coraor N, Ananda G, Lazarus R, Mangan M, Nekrutenko A, Taylor J: **Galaxy: a web-based genome analysis tool for experimentalists.** *Curr Protoc Mol Biol* 2010, **Chapter 19**:Unit 19.10.1–21.

2. Quinlan AR, Hall IM: **BEDTools: a flexible suite of utilities for comparing genomic features.** *Bioinformatics* 2010, **26**:841–842.

3. Li H, Handsaker B, Wysoker A, Fennell T, Ruan J, Homer N, Marth G, Abecasis G, Durbin R: **The Sequence Alignment/Map format and SAMtools.** *Bioinformatics* 2009, **25**:2078–2079.

4. Dajnoki A, Mühl A, Fekete G, Keutzer J, Orsini J, Dejesus V, Zhang XK, Bodamer OA: **Newborn screening for Pompe disease by measuring acid alpha-glucosidase activity using tandem mass spectrometry**. *Clin Chem* 2008, **54**:1624–1629.
